# Effects of Extraction Strategies on Yield, Physicochemical and Antioxidant Properties of Pumpkin Seed Oil

**DOI:** 10.3390/foods12183351

**Published:** 2023-09-07

**Authors:** Tianyuan Hu, Li Zhou, Fan Kong, Shu Wang, Kunqiang Hong, Fenfen Lei, Dongping He

**Affiliations:** 1 College of Food Science and Engineering, Wuhan Polytechnic University, Wuhan 430023, China; hutianyuan2000@126.com (T.H.); zhouli910228@163.com (L.Z.); kongfan2023@163.com (F.K.); hongkq@whpu.edu.cn (K.H.); hedp123456@163.com (D.H.); 2 Key Laboratory of Edible Oil Quality and Safety for State Market Regulation, Wuhan 430023, China; 3 Grain and Oil Resources Comprehensive Exploitation and Engineering Technology Research Center of State Administration of Grain, Wuhan 430023, China; wangshu8532@163.com; 4 Wuhan Institute for Food and Cosmetic Control, Wuhan 430023, China

**Keywords:** pumpkin seed oil, microwave pretreatment, supercritical fluid extraction, bioactive compounds, antioxidant properties

## Abstract

This study investigated the effects of three extraction methods, including cold pressing (CP), microwave pretreatment pressing (MP), and supercritical fluid extraction (SFE), on the yield, physicochemical properties, bioactive compounds content, and antioxidant properties of pumpkin seed oil (PSO). Furthermore, the correlation between bioactive compounds and the antioxidant properties of PSO was determined. The results revealed that the yield of PSO extracted using the three methods was in the order of SFE > MP > CP. Additionally, the PSO generated by SFE showed the highest unsaturated fatty acid content, followed by MP and CP. Additionally, MP-PSO exhibited the highest acid value and saponification value, while SFE-PSO displayed the highest moisture content, peroxide value, and iodine value. Moreover, the PSO generated by MP demonstrated superior antioxidant properties compared to that of PSOs from CP and SFE in the oxidation induction, DPPH, FRAP, and ABTS tests. Finally, the correlation analysis revealed that specific types of bioactive compounds, such as β-sitosterol and γ-tocopherol, were highly correlated with the antioxidant properties of PSOs. Consequently, this study provides comprehensive knowledge regarding PSO extraction, physicochemical properties, bioactive compound extraction, and the correlated antioxidant properties.

## 1. Introduction

Pumpkin is a vining herbaceous plant belonging to the Cucurbita genus in the Cucurbitaceae family, which comprises over 119 genera and 825 species [1]. The flesh of pumpkin can be used to create various food products, such as dried fruits, pies, soups, juices, cakes, and so on, whereas pumpkin seeds are mainly consumed as flavored snacks through roasting. Pumpkin seeds are rich in protein, lipids, minerals, sugar, fiber, starch, and bioactive compounds, making them an excellent source of food products [2]. Specifically, pumpkin seeds contain over 30.0% lipids in their composition, with the main fatty acids identified as oleic acid, stearic acid, palmitic acid, and linoleic acid, among which oleic acid (21–47%) and linoleic acid (36–61%) are the richest fatty acids [3]. Pumpkin seed oil (PSO), extracted from the pumpkin seeds, has attracted considerable attention in recent years due to the potential health benefits of unsaturated fatty acids and micro-nutritive and antioxidant compounds, including phytosterol, tocopherols, squalene, phenolic acid, vitamins, and carotenoids [4]. Besides cooking, it can be used as a functional food to treat and prevent cardiovascular and cerebrovascular diseases and prostate diseases [5]. PSO has been recognized as a high-quality vegetable oil for both medicine and food in the world. The processing technology of PSO had significant effect on its composition and content of lipid companions, which further determined the nutritional and functional activity of PSO [6]. Exploring the effects of different processing techniques on the nutritional components of PSO is of great significance to maximize the nutritional and functional properties of PSO.

Among vegetable oil extraction strategies, the cold pressing extraction method is highly regarded, especially for high-end oil products, such as olive oil, due to the advantages, including light color, better taste, low free fatty acid content, low peroxide value, and its high retention of bioactive compounds [7]. However, profit-driven vegetable oil manufacturers often criticize cold pressing results from its low oil yield. Microwave pretreatment on oil seeds has been reported as an efficient strategy to improve the oil yield and release of bioactive compounds during extraction [8]. Additionally, supercritical fluids exhibit extraordinary extraction characteristics on seed oils when subjected to a gas-to-liquid phase extraction medium under high pressure and vacuum near or above the critical point, resulting in higher oil extraction yield [9]. Indeed, the supercritical fluid extraction method has been widely employed in vegetable oil extraction, including PSO, due to its benefits, such as high-pressure and low-temperature process conditions, high oil yield, and high retention of bioactive compounds [10]. Rezig et al. [11] evaluated the chemical composition and the bioactive compounds of PSOs using both cold pressing and solvent extraction methods. Further comparison of these extraction methods for PSO is insufficient, which is crucial for the efficient production of high-quality PSO.

The antioxidant properties of oils are determined by the fatty acid profile and the presence of antioxidant compounds. Saturated fatty acids (e.g., stearic acid and palmitic acid) generally exhibit stronger antioxidant activity than unsaturated fatty acids (e.g., oleic acid, linoleic acid, and linolenic acid) [12]. Moreover, bioactive compounds, such as tocopherols, phytosterols, polyphenols, squalene, and carotenoids, strongly contribute to the antioxidant properties of vegetable oils [13]. The oxidation induction time, which measures the time from introducing hot air into the oil until the release of electrically conductive oxidation components, is commonly used to evaluate the antioxidant capacity of vegetable oils [14]. Additionally, physical and chemical methods are usually applied to measure the vegetable oils’ antioxidant properties. For example, the 2,2′-diphenyl-1-picrylhydrazyl (DPPH) radical assay is used to measure the radical scavenging activity of antioxidant agents, the ferric reducing antioxidant power (FRAP) assay is employed to assess the antioxidative capacity of antioxidants, and the 2,2′-azinobis-3-ethylbenzothiazoline-6-sulfonic acid (ABTS) assay is used to evaluate the ability to clear ABTS▪+ free radicals [15]. Although the antioxidant properties of vegetable oil bioactive compounds have been extensively studied by various research groups, there is a lack of comparisons between the antioxidant capacity of PSOs generated from different processes and the correlation analysis between specific types of bioactive compounds and the antioxidant properties.

In this study, the extraction strategies of PSO using three methods, cold pressing (CP), microwave pretreatment pressing (MP), and supercritical fluid extraction (SFE), were investigated to aid in the production of PSO with rich bioactive compounds and better antioxidant capacity. Additionally, the oil yield and physicochemical properties of PSOs extracted by these three methods were compared. Further, the relationship between PSO antioxidant properties and bioactive compounds was determined. The specific objectives of this study were as follows: (1) to investigate the application of CP, MP, and SFE for extracting PSO; (2) to compare the yield, physicochemical properties, content of bioactive compounds, and antioxidant properties of PSO obtained through CP, MP, and SFE; and (3) to examine the correlation between bioactive compounds present in PSOs and their antioxidant properties, including oxidation induction, DPPH, FRAP, and ABTS. These will clarify the impact of processing technologies on the physicochemical properties and chemical composition of PSO and promote the development of a functional PSO industry.

## 2. Materials and Methods

### 2.1. Materials

Pumpkin seeds with husks, harvested at the commercial maturity stage, were obtained from Powdery Health Industry Co., Ltd., Jinmen, China) and used as the raw material for PSO extraction. Standards of fatty acid esters, triacylglycerol; α-, β-, γ- and δ-tocopherols (purity > 95%); squalene; 5α-cholestane; campesterol; stigmasterol; β-sitosterol; Bis(trimethylsilyl)trifluoroacetamide (BSTFA) + Trimethylchlorosilane (TMCS); DPPH, 2,4,6-Tris (2-pyridyl)-s-triazine (TPTZ); ABTS; and fluorescein sodium salt were purchased from Sigma-Aldrich Chemical Co., Ltd. (Shanghai, China). The 6-Hydroxy-2,5,7,8-tetramethylchroman-2-carboxylic acid (Trolox) was obtained from TCI Development Co., Ltd. (Shanghai, China). All other chemicals were of analytical grade and were purchased from Sinopharm Chemical Regent (Shanghai, China).

### 2.2. PSO Extraction

Three types of PSOs were generated for this study using CP, MP, and SFE. Firstly, complete CP oil was produced using a single screw oil press (M222/20F, Miramar Nowa Wieś, Dziećmorowice, Poland), with the temperature of the generated oil tested to be 45 ± 1 °C. Secondly, the pumpkin seeds were spread in a single layer in a Petri dish (12 cm diameter) and exposed to a microwave oven (PM2302, Midea, Guangdong, China) at 500 W for 10 min. After microwave treatment, the pumpkin seeds were crushed to produce oil using the same procedure as the complete cold-pressing method mentioned above. Lastly, SFE was employed to obtain PSO using an SFE Bio-Botanical extraction system from Gaoke Pharmaceutical Equipment Co., Ltd. (GKSFE 120-50-05, Nantong, China). Specifically, 150 g of pumpkin seed powder was placed in a 2 L extraction vessel, and carbon dioxide with a purity of 99.99% was injected into the SFE apparatus at a flow rate of 0.5 L/min under a pressure of 35 MPa for 3.5 min. The SFE process was carried out for 1 h at a temperature of 50 °C and pressure of 9 MPa. Subsequently, the extraction vessel was depressurized, and the oil was collected from the separating vessel. All the three types of PSOs were sealed and stored at 4 °C for downstream analysis. The yield of PSO was calculated as the percentage ratio of the mass of oil to the mass of the pumpkin seeds, using the following formula:

Extraction yield of pumpkin seed oil (%) = ((mass of the extracted oil)/(mass of pumpkin seeds)) × 100

### 2.3. Determination of PSO Physicochemical Properties

The moisture contents of the PSO samples were determined using a V20 Volumetric Karl Fischer Titrator (Mettler-Toledo, Columbus, OH, USA), following the method described by Zhou et al. [16] and were expressed as percentage. The acid values in the oil samples were tested following the AOCS official method, Cd 5a-40, and were expressed as mg KOH/g oil. The peroxide values were determined using AOCS method Cc 13k-13 and were expressed as mmol/kg. The Iodine values in the oil samples were measured following the AOCS official method, Cd 1d-92, and were expressed as mg I_2_/g oil. The saponification values of the oil samples were detected following the AOCS official method, Cd 3-25, and were expressed as mg KOH/g oil.

### 2.4. Determination of PSO Fatty Acid Profile

The fatty acid profiles of the PSO samples were prepared by transesterification oils into fatty acid methyl esters using methanol and potassium hydroxide according to the ASTM D-1983 standard [17] method and analyzed by GC-MS (450-GC/240-MS, Varian, Santa Clara, CA, USA) following the method described by Gao et al. [12]. The content of each fatty acid was presented in percent of the peak area of all fatty acids.

### 2.5. PSO Bioactive Compounds

#### 2.5.1. Determination of Total Polyphenol Content

The total polyphenol content in PSOs was determined following a modified procedure described by Fuentes et al. [18]. Briefly, methanolic extracts of PSO samples (5 mL) were mixed with 0.5 mL of Folin–Ciocalteu reagent and kept in the dark for 3 min. Afterward, 1 mL of 10% sodium carbonate was added, and the tubes were incubated in the dark for 2 h. The absorbance was measured at 765 nm using a UV-Vis spectrophotometer, and the quantitative results were calculated using an analytical curve of gallic acid. The total polyphenol content was expressed as milligrams of gallic acid equivalents (GAE) per kilogram of PSO sample (mg/kg).

#### 2.5.2. Determination of Phytosterol and Squalene Content

The determination of phytosterol and squalene compounds in PSOs was carried out following the method described by Olmo-García et al. [19] with slight modifications, using GC-MS (7890A, Agilent Technologies, Folson, CA, USA) equipped with a capillary column (HP-5MS, 30 μm, 30 m × 0.25 mm). Helium was used as the carrier gas at a flow rate of 1.5 mL/min. The GC column temperature was programmed from 60 to 300 °C at a rate of 3 °C/min and held for 10 min. The ion source temperature was 230 °C, and the quadrupole temperature was 150 °C. The identification of compounds was confirmed by the retention time lock (RTL) method and RTL Adams database.

#### 2.5.3. Determination of Tocopherol Content

The determination of tocopherols in PSO was conducted with slight modifications to the method described by Constantin et al. [20]. Briefly, PSO samples were saponified before the hexane-soluble compounds (containing tocopherols) were collected for drying by nitrogen gas. The tests were conducted using a HPLC (2695 Waters, Milford, MA, USA) equipped with a reversed-phase Nucleosil 50-5 C18 column and an RF-535 fluorescence detector (Shimadzu, Tokyo, Japan). The HPLC system was operated with an excitation wavelength of λ = 290 nm and an emission wavelength of λ = 330 nm. The relative retention time and maximum absorption values at the given relative retention time were used for the identification of tocopherols in the oil samples.

### 2.6. PSO Antioxidant Properties

The oxidation induction of PSOs was determined using a Rancimat 743 apparatus (Metrohm, Herisau, Switzerland), following the ISO 6885 method. Briefly, approximately 3 g of the PSO sample were accurately weighed and added into the test tubes of the Rancimat apparatus with the temperature set to be 120° and an oxygen gas flow of 20 dm^3^/h. ABTS and DPPH radical cation scavenging capacity were conducted following the procedure described by Gao et al. [15]. The absorbance value of the mixture of oil sample and ABTS or DPPH after the reaction was measured at 734 nm and 517 nm using an UV-Vis spectrophotometer, respectively. For the FRAP assay, 0.3 mL of acetonic extracts of the PSO samples and 2 mL of the FRAP reagent were reacted at 37 °C for 10 min. The maximum absorbance values of the solutions were measured at 595 nm. An analytical curve using Trolox as a standard was constructed. The antioxidant capacity was expressed as micromoles (μmol) of Trolox equivalents per 100 g of the PSO sample.

### 2.7. Statistical Analysis

The analyses were performed in triplicate, and the data are presented as mean ± SD (*n* = 3). Statistical analyses were conducted using the Statistical Package for the Social Sciences (SPSS) version 25.0 (IBM Corp., Armonk, NY, USA). Specifically, the normality and homogeneity of the variances were checked using the Shapiro–Wilk and Levene tests, respectively, before ANOVA. Meanwhile, one-way ANOVA and Duncan post hoc tests were employed to compare the differences in oil yield, bioactive compound contents, and antioxidant indicators. Additionally, multiple correlation analysis was utilized to determine the correlation between the antioxidant properties and the bioactive compounds. Significant differences were reported at the 95% confidence interval (*p*-value < 0.05). This section may be divided by subheadings. It should provide a concise and precise description of the experimental results, their interpretation, as well as the experimental conclusions that can be drawn.

## 3. Results and Discussion

### 3.1. Yield and Fatty Acid Profile of PSO

**Figure 1:**
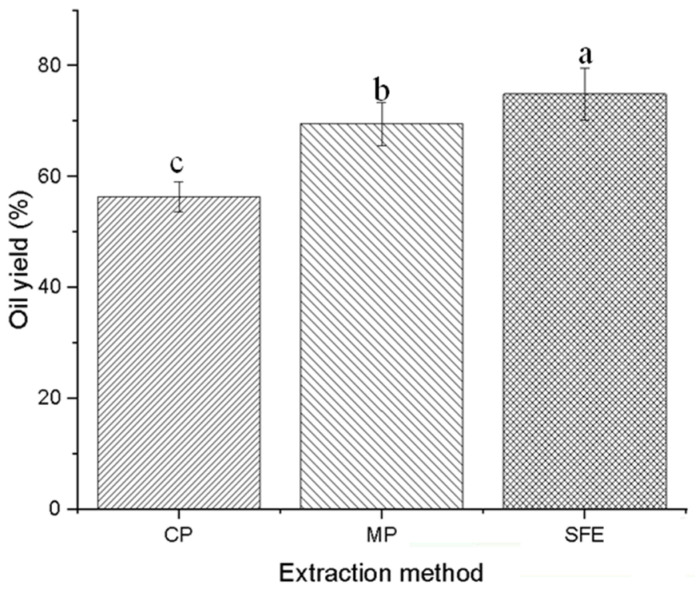
The yield of pumpkin seed oil by cold pressing, microwave-pretreatment pressing, and supercritical fluid extraction. Within each extraction method, different letters denote statistically significant differences for *p* < 0.05.

The yield of PSO extracted by CP, MP, and SFE was 56.4%, 69.5%, and 74.9%, respectively (Figure 1). This finding indicates that microwave-pretreatment significantly promotes the extraction of oil compared to the direct cold-pressing method (independent *t*-test, *p*-value < 0.05). Microwave pretreatment enhances vegetable oil yield by rupturing the oil seed cell membrane and promoting mass transfer [21]. Moreover, SFE exhibited the highest yield among the three methods. SFE strategy has a higher diffusivity and lower viscosity compared to liquid solvent extraction, resulting in increased efficiency in mass transfer [22]. These results are similar as to the values reported by other researchers: Jiao et al. [23] reported that microwave-assisted enhanced pumpkin seed oil yield, reaching 64.7%, while Bernardo-Gil et al. [24] reported that the supercritical fluid extraction generated pumpkin seed oil with a yield near 80% when converted to mass ratio at premium conditions.

Table 1 presents the fatty acid profile of PSOs extracted using CP, MP, and SFE. Generally, the highest proportions of fatty acids in PSOs are oleic acid and linoleic acid, comprising approximately 35% and 39%, respectively. These are followed by stearic acid (around 14%) and palmitic acid (around 11%). Notably, PSO obtained through the CP method exhibited the highest saturated fatty acid content and the lowest unsaturated fatty acid content, while oil extracted through the SFE strategy showed the lowest saturated fatty acid content and the highest unsaturated fatty acid content (Table 1). The SFE extraction strategy operates on the principle of dissolving vegetable oil in a liquid with complete contact under supercritical conditions, which leads to a higher yield of unsaturated fatty acids due to their relatively low melting point and high mobility [25]. Similarly, PSO extraction with microwave pretreatment demonstrated lower levels of saturated fatty acids and higher levels of unsaturated fatty acids compared to direct cold pressing oil. Microwave pretreatment generates internal heat within the oil-containing cells and transfers it to the outer parts of the seeds, facilitating the efficient transfer of unsaturated fatty acids due to their lower viscosity [26], resulting in a higher ratio of unsaturated to saturated fatty acids. The results of this study are similar to the values reported by Ali et al. [27], in which the main unsaturated fatty acids oleic acid and linoleic acid content were tested to be 33.9% by CP and 33.0% after a 12 min microwave pretreatment, and 37.9% by CP and 36.8% after a 12 min microwave pretreatment, respectively. Overall, both MP and SFE strategies enhance the yield of PSO and contribute to the release of unsaturated fatty acids from seeds.

### 3.2. Physicochemical Properties of PSOs

Among the PSOs extracted by the three methods, SFE PSO exhibited the highest moisture content (1.05%), followed by CP (0.28%) and MP (0.11%) (Table 2). The solubility of moisture in supercritical fluid is greatly influenced by pressure and temperature. The moisture content was able to reach 5.59% in a study regarding the measurement of water solubility in supercritical CO_2_ fluid [28]. The low moisture content in MP oil could be attributed to the heat generated in microwave pre-treatment evaporating moisture from the oil at a high temperature. MP PSO showed a higher acid value compared to CP and SFE oils, while SFE oil exhibited the highest peroxide value, followed by MP and CP oils. The heat generated during microwave pretreatment can promote oil oxidation [29], leading to higher acid and peroxide values than CP-PSO. Additionally, supercritical CO_2_ has some solubility for free fatty acids and peroxides [30], which aligns with the results in this study. The iodine value represents the degree of unsaturation of lipids and estimates the content of unsaturated fatty acids [31]. SFE PSO showed a significantly higher iodine value than CP and MP oils (Table 2), which aligns with the data presented in Table 1, in which SFE oil exhibited higher levels of unsaturated fatty acids. The saponification value reflects the purity and molecular weight of triglycerides in lipids [32]. The saponification values of PSOs extracted through these three methods are statistically similar, indicating that the average weight of triglycerides extracted through CP, MP, and SFE is indistinguishable. These findings align with the statements by other researchers where MP vegetable oil displayed a higher acid value, peroxide value, saponification value, and a lower moisture content and iodine value compared to CP oil [7]. Meanwhile, SFE vegetable oil exhibited a high moisture content and high peroxide value but a lower acid value and iodine value compared to CP oil [9].

### 3.3. Contents of Bioactive Compounds in PSOs

**Figure 2:**
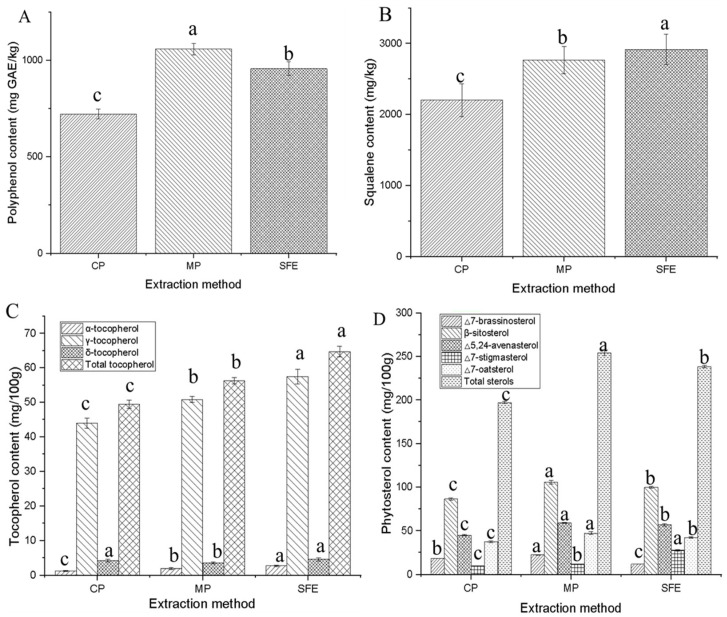
Contents of bioactive compounds in pumpkin seed oils: (**A**) polyphenol, (**B**) squalene, (**C**) tocopherol, (**D**) phytosterol. Within each extraction method, different letters denote statistically significant differences for *p* < 0.05.

Generally, MP-PSO exhibited higher content of polyphenols, squalene, tocopherols, and phytosterols compared to CP oil (independent *t*-tests, all *p*-values < 0.05) (Figure 2). Similarly, SFE-produced PSO showed higher contents of polyphenols, squalene, tocopherols, and phytosterols compared to CP oil (independent *t*-tests, all *p*-values < 0.05). However, when comparing SFE and MP oils, the superiority of SFE in extracting bioactive compounds from PSO is not distinct. SFE oil demonstrated higher levels of squalene and tocopherols but lower levels of polyphenols and phytosterols compared to MP oil (independent *t*-tests, all *p*-values < 0.05) (Figure 2). The tocopherol chromatogram of different PSOs is presented in the Supplementary Material (Figure S1). Microwave pre-treatment offers several advantages in the extraction of bioactive compounds from oil-bearing materials, including shorter extraction times, more efficient heating, lower energy consumption, and reduced thermal gradients [33]. Rezig et al. [11] found a higher tocopherol content in the cold-pressed PSO (cultivar C. maxima Bejiaoui) compared to that of PSO extracted with solvent. Šamec et al. [5] reported that the α-tocopherol, γ-tocopherol, and δ-tocopherol content in PSO extracted by different techniques were 37.5 ug/g, 383 ug/g, and not detected via cold pressing (Tunisia), 61.3 uk/g, 492.8 ug/g, and 35.3 ug/g via SFE from pumpkin from the USA. Phytosterol content in PSO extracted by cold pressing was 782.1–805.2 mg/100 g [34], in PSO extracted by SFE CO_2_ was 294 mg/100 g oil [35]. Total polyphenol contents of commercially available cold-pressed PSOs in Poland were 53.67−184.6 mg CAE/kg oil [36]. Researchers have widely reported increased levels of squalene; α-, β-, γ-tocopherols; and phytosterols in vegetable oils with microwave pre-treatment [37]. SFE also has several advantages, including its environmentally friendly nature, high efficiency, availability at high purity levels, and high solubility of bioactive compounds in vegetable oil extraction [38]. Altogether, the MP extraction method demonstrated superiority in retaining bioactive compounds, including polyphenols, tocopherols, squalene, and phytosterols, in PSO compared to CP. The effect of the SFE extraction method on PSO in terms of bioactive compounds is influenced by the specific extraction conditions employed.

### 3.4. Antioxidant Properties of PSOs

**Figure 3:**
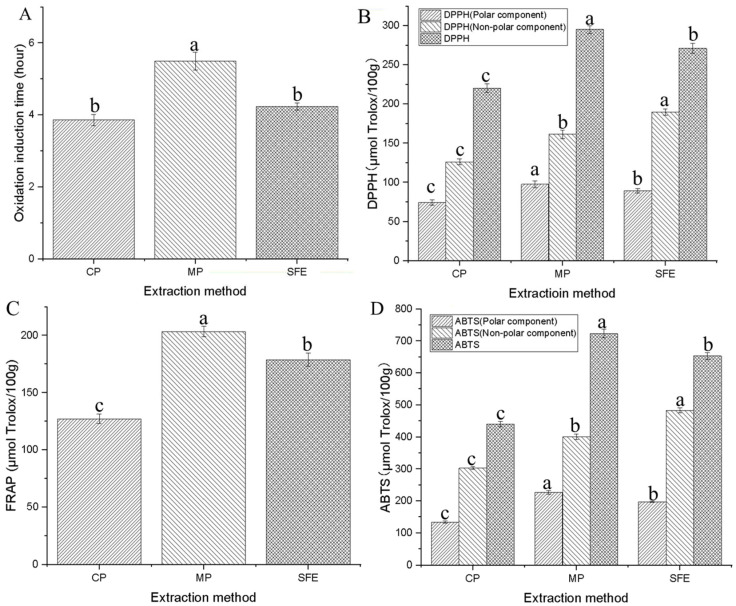
Antioxidant capacity of pumpkin seed oils: (**A**) oxidation induction time, (**B**) DPPH, (**C**) FRAP, (**D**) ABTS. Within each extraction method, different letters denote statistically significant differences for *p* < 0.05.

The results indicate that the antioxidant properties of PSO extracted using the three methods follow the order of MP > SFE > CP (one-way ANOVA, all *p*-values < 0.05) (Figure 3). Specifically, MP-PSO exhibited higher resistance to hot air oxidation compared to CP-PSO, while SFE oil showed a slightly higher value in oxidation induction compared to CP oil (Figure 3A). These findings are consistent with a broad range of similar studies. For instance, Kaseke et al. [39] reported that a 120 s microwave pre-treatment at 261 W significantly improved DPPH and FRAP values while enhancing the content of phytosterols in pomegranate seed oil extraction. Similarly, in a study on microwave pre-treatment-assisted extraction of rice bran oil, microwave treatment greatly improved DPPH, FRAP, and ABTS values [40]. The enhanced antioxidant properties of PSO can be attributed to the promotion of the release of natural antioxidant compounds, including tocopherols, phytosterols, and polyphenols, through microwave pre-treatment [41]. Additionally, SFE has been reported to enhance the content of these bioactive compounds in vegetable oils [42]. In fact, the results presented in Section 3.3 concluded that the content of natural antioxidant compounds, including tocopherols, phytosterols, and polyphenols, in PSO extracted using the three methods follows the order of MP > SFE > CP, which aligns with the findings in this section.

### 3.5. Correlation Analysis of PSO Bioactive Compounds with Antioxidant Stability

Table 3 displays a low correlation between the oxidation induction time of PSO and the content of bioactive compounds (polyphenols, tocopherols, phytosterols, and squalene). This can be attributed to the fact that the oxidation induction time of lipids is influenced not only by the bioactive compounds but also by the fatty acid profile and the potential synergistic effects between bioactive compounds and the fatty acid profile [43]. However, significant correlations were observed between the polyphenol content of PSO and the FRAP values (*p*-value < 0.05), with a correlation coefficient of 0.769 (Table 3). Furthermore, the correlation between the polyphenol content of PSO and the DHHP value (0.824) and ABTS value (0.810) was highly significant (*p*-value < 0.01) (Table 3). This suggests that phenolic compounds play a crucial role in the antioxidant properties of PSO. Additionally, the α-tocopherol content of PSO showed correlations with DPPH and ABTS values (*p*-value < 0.05), while the contents of β-sitosterol, total sterols, γ-tocopherol, and total tocopherol exhibited extremely or highly significant correlations with the DPPH and ABTS values in PSO (*p*-values < 0.01) (Table 3). These findings indicate that the antioxidant properties of PSO are correlated with specific bioactive compounds, including total phenols, certain types of phytosterols, and tocopherols.

## 4. Conclusions

In conclusion, vegetable oil extraction strategies such as CP, MP, and SFE can be effectively employed for PSO extraction. Among these methods, SFE demonstrated the highest yield of PSO, along with the highest content of unsaturated fatty acids and moisture content. On the other hand, the MP method resulted in the highest acid value and peroxide value. Additionally, this method yielded PSO with the highest levels of phytosterol and polyphenol, while the SFE method generated PSO with the highest tocopherol and squalene contents. Furthermore, the MP PSO exhibited the strongest antioxidant properties compared to the oils extracted using the other two methods. Notably, specific bioactive compounds including polyphenol, α-tocopherol, β-sitosterol, and γ-tocopherol showed a strong correlation with the antioxidant ability of PSO. Altogether, the MP PSO demonstrated favorable results in terms of indicators such as oil yield, physicochemical properties, bioactive compounds content, and antioxidant properties. Further research should concentrate on the optimization of operation parameters of microwave pre-treatment on pumpkin seeds, which possesses relatively high quality of oil. Additionally, given the remarkable ability of MP to release bioactive compounds, such as tocopherol, phytosterol, squalene, and polyphenol from PSO, the preservation of these bioactive compounds during the oil refining process should be taken into consideration.

## Figures and Tables

**Table 1:** Fatty acid profile of pumpkin seed oils extracted by cold pressing, microwave-pretreatment pressing, and supercritical fluid extraction.

Extraction Method	CP	MP	SFE
Myristic acid (C14:0)	ND	ND	0.100 ± 0.04 ^a^*
Palmitic acid (C16:0)	11.83 ± 0.04 ^a^	11.30 ± 0.03 ^b^	10.74 ± 0.07 ^c^
Stearic acid (C18:0)	15.48 ± 0.12 ^a^	14.45 ± 0.06 ^b^	13.30 ± 0.09 ^c^
Oleic acid (C18:1)	34.16 ± 0.10 ^b^	35.38 ± 0.06 ^a^	35.38 ± 0.07 ^a^
Linoleic acid (C18:2)	37.88 ± 0.03 ^c^	38.27 ± 0.08 ^b^	39.83 ± 0.05 ^a^
Linolenic acid (C18:3)	0.65 ± 0.03 ^a^	0.59 ± 0.01 ^b^	0.66 ± 0.02 ^a^
SFA	27.31 ± 0.09 ^a^	25.76 ± 0.06 ^b^	24.14 ± 0.10 ^c^
USFA	72.69 ± 0.07 ^c^	74.24 ± 0.08 ^b^	75.86 ± 0.06 ^a^

* Values are means ± standard deviations. The superscript letters indicate the statistical differences in lines at significance level of 5%. ND: Undetected.

**Table 2:** Physicochemical properties of pumpkin seed oils extracted with different methods.

Extraction Method	CP	MP	SFE
Moisture content (%)	0.28 ± 0.03 ^b^*	0.11 ± 0.01 ^c^	1.05 ± 0.04 ^a^
Acid value (mg (KOH)/g)	0.61 ± 0.04 ^c^	0.90 ± 0.07 ^a^	0.78 ± 0.06 ^b^
Peroxide value (mmol/kg)	1.23 ± 0.08 ^c^	1.58 ± 0.07 ^b^	2.11 ± 0.10 ^a^
Iodine value (mg I_2_/g)	129.36 ± 2.45 ^b^	128.97 ± 3.83 ^b^	133.21 ± 1.24 ^a^
Saponification value (mg (KOH)/g)	188.3 ± 2.14 ^a^	190.2 ± 2.57 ^a^	188.9 ± 1.48 ^a^

* Values are means ± standard deviations. The superscript letters indicate the statistical differences in lines at significance level of 5%.

**Table 3:** Correlation analysis of pumpkin seed oil bioactive compounds with antioxidant stability.

	Oxidation Induction Time	DPPH (Polar)	DPPH (Non-Polar)	DPPH	ABTS (Polar)	ABTS (Non-Polar)	ABTS	FRAP
Polyphenol	0.614	0.921 **	0.435	0.824 **	0.956 **	0.424	0.810 **	0.769 *
Δ 7-brassinosterol	0.474	0.213	−0.562	0.183	0.175	−0.570	0.152	0.186
β-sitosterol	0.603	0.376	0.906 **	0.401	0.445	0.886 **	0.491	0.365
Δ 5,24-avenasterol	0.162	0.378	0.417	0.349	0.306	0.368	0.270	0.450
Δ 7-stigmasterol	−0.113	0.286	0.483	0.260	0.313	0.442	0.272	0.303
Δ 7-oat sterol	0.530	0.081	0.209	0.055	0.049	0.151	0.074	0.977
Total sterols	0.621	0.517	0.843 **	0.579	0.527	0.836 **	0.636	0.545
α-tocopherol	0.166	0.242	0.687 *	0.265	0.271	0.658 *	0.324	0.263
γ-tocopherol	0.174	0.648	0.868 **	0.670	0.676	0.828 **	0.729	0.669
δ-tocopherol	−0.376	−0.511	0.271	−0.485	−0.478	0.280	−0.412	−0.487
Total tocopherol	0.108	0.596	0.953 **	0.620	0.626	0.974 **	0.582	0.618
Squalene	0.269	0.451	0.567	0.466	0.570	0.564	0.604	0.565

* Indicates significant correlation (*p* < 0.05), ** indicates extremely significant correlation (*p* < 0.01).

## Data Availability

No new data were created or analyzed in this study. Data sharing is not applicable to this article.

## Supplementary Materials

The following supporting information can be downloaded at: https://www.mdpi.com/article/10.3390/foods12183351/s1, Figure S1: The tocopherol chromatogram of different pumpkin seed oils.
