# Supplementary figures and images for: Effects of Extraction Strategies on Yield, Physicochemical and Antioxidant Properties of Pumpkin Seed Oil
Source: Foods. 2023 Sep 7;12(18):3351. doi: 10.3390/foods12183351 (PMC10529489; doi:10.3390/foods12183351)

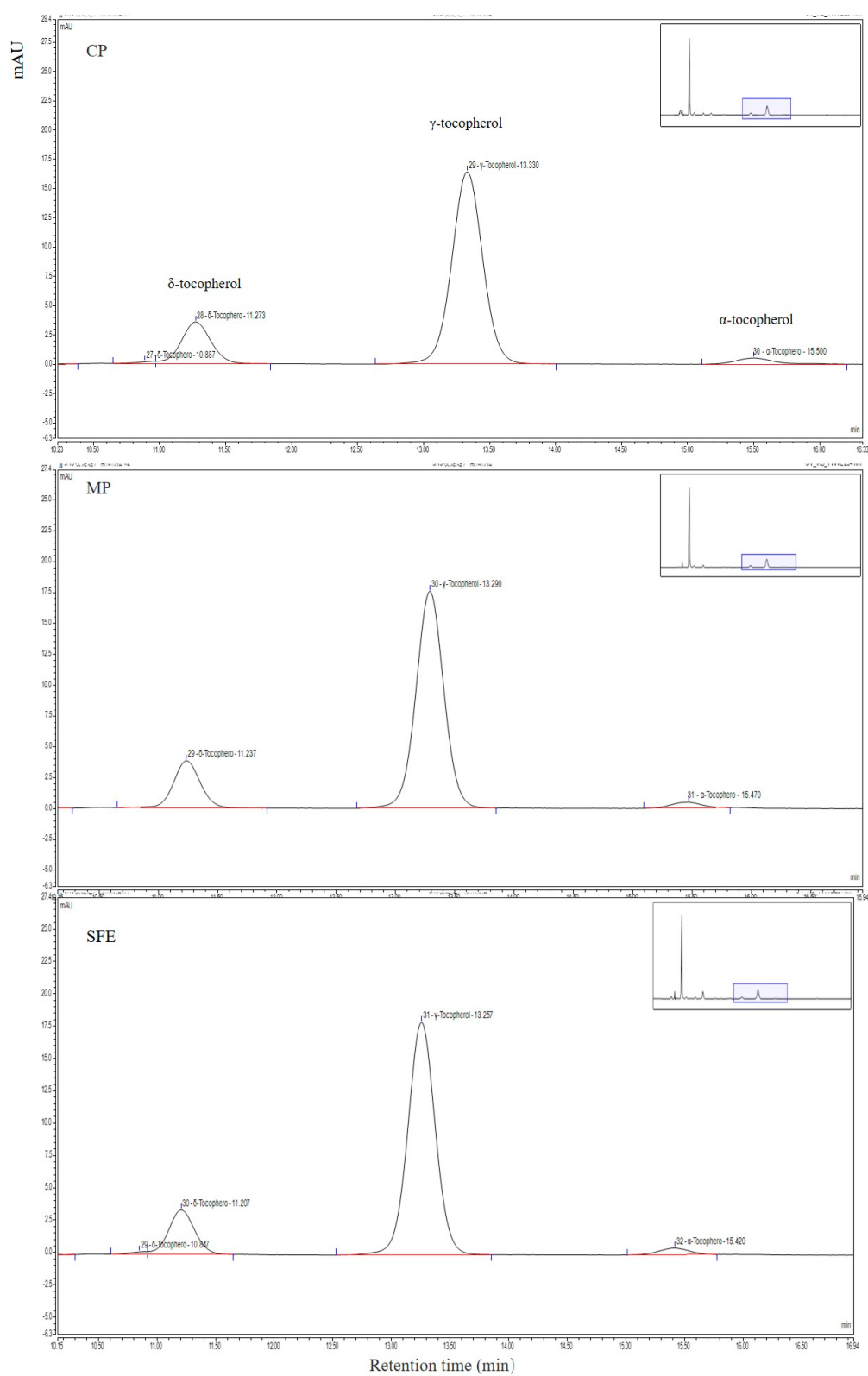

Figure S1. The tocopherol chromatogram of different pumpkin seed oils.

Supplement: Supplementary file 1 [file foods-12-03351-s001.zip › foods-2591650-supplementary.pdf]
